# To fast or not to fast

**DOI:** 10.1192/j.eurpsy.2021.1802

**Published:** 2021-08-13

**Authors:** G. Marinho, C. Cotta, S. Vieira, J. Peta, M. Marguilho

**Affiliations:** 1 Clinica 6, CHPL, Lisbon, Portugal; 2 Psiquiatria De Ligação, CHLC, Lisboa, Portugal; 3 Psychiatry, Centro Hospitalar Psiquiátrico de Lisboa, Lisboa, Portugal; 4 Clínica 5, Centro Hospitalar Psiquiátrico de Lisboa, Lisboa, Portugal

**Keywords:** Circadian rhythm, bipolar disorder, ramadan, fasting

## Abstract

**Introduction:**

Ramadan happens in the ninth month of the Muslim lunar calendar. The cycle of the sun marks the beginning and the end of fasting. Its duration varies depending on the season: approximately 18 h in the summer to approximately 12 h during winter. The obligation to eat only during the night leads to an important change in the circadian rhythm There are certain psychiatric illnesses wherein people are very sensitive to this circadian disruption, bipolar disorder in particular. We know that a regulated circadian rhythm with adequate sleep are essential for symptom regulation and mood stability, with the risk of relapse or worsening symptoms. Additionally, some medications have to be maintained at a specific therapeutic index, namely lithium, a common mood stabilizer used to treat bipolar disorder.

**Objectives:**

To review the impact of Ramadan on patients with bipolar disorder

**Methods:**

Pubmed and Google Scholar search using the keywords Bipolar disorder, Ramadan, circadian rhythm, fasting, sleep deprivation

**Results:**

All physiologic parameters are influenced by the circadian rhythm, which is influenced in its turn by the food rhythm. Studies on the effects of Ramadan on mood and mental health in the general population provide contradicting evidence. The inability to take medications during the day, dehydration and other somatic changes that necessitate dosing modification may lead to psychiatric symptom exacerbation.

**Conclusions:**

Patients with bipolar disorder might be particularly sensitive to circadian rhythm disturbances and could require increased monitoring of their symptoms during this month.

**Disclosure:**

No significant relationships.

